# Pattern of impairments and late diagnosis of autism spectrum disorder among a sub-Saharan African clinical population of children in Nigeria

**DOI:** 10.1017/gmh.2016.30

**Published:** 2017-03-21

**Authors:** M. A. Bello-Mojeed, O. O. Omigbodun, M. O. Bakare, A. O. Adewuya

**Affiliations:** 1 Child and Adolescent Mental Health Service Centre, Federal Neuro-Psychiatric Hospital, Lagos, Nigeria; 2 Childhood Neuropsychiatric Disorders Initiatives, Nigeria; 3 Department of Psychiatry, University College Hospital, Ibadan, Nigeria; 4 College of Medicine, Enugu State University of Science and Technology (ESUT), Enugu, Nigeria; 5 Child and Adolescent Unit, Federal Neuro-Psychiatric Hospital, New Haven, Enugu, Nigeria; 6 Department of Behavioural Medicine, Lagos State University College of Medicine, Lagos, Nigeria

**Keywords:** Africa, ASD, diagnosis, identification, impairment, Nigeria

## Abstract

**Background.:**

Autism spectrum disorder (ASD) is a complex group of neurodevelopmental disorders. Studies conducted among Africans living outside the continent indicate that African children are more likely than Caucasian children to have a late diagnosis of ASD. There is a dearth of information on this topic among children with ASD living in Africa.

**Methods.:**

To determine the pattern of impairments and age at diagnosis in ASD, sixty Nigerian children with a diagnosis of ASD were recruited from a neurodevelopmental clinic. DSM-V criteria were used to make a diagnosis of ASD, while a symptom checklist for ASD was used to determine the pattern of impairments in ASD.

**Results.:**

Ages of the children ranged from 2 to 17 years with a mean age of 9.45 ± 4.33 years with the majority of them (75%) being 12 years or younger. All the children (100%) with ASD exhibited poor eye contact, difficulty in mixing with other children and inability to consistently respond to his/her name. More than a half of them (55%) lack verbal communication. Impairments that were uncommon are in the areas of object attachment (20.0%), odd postures (26.7%) and inappropriate facial expression (30.0%). Mean age at the observation of ASD features was 17.0 ± 6.7 months. Mean age at diagnosis of ASD was 9.00 ± 4.30 years. The mean time lag from a parental concern of ASD features to seeking specialist care was 85 months and to diagnosis was 91 months.

**Conclusions.:**

Core symptoms/impairments of ASD are present in Nigerian children but a late diagnosis is common.

## Introduction

Autism spectrum disorder (ASD) is a complex lifelong, disabling, neurodevelopmental group of disorders that afflict a child in an early developmental period. Although impairments in ASD affect virtually all aspects of the child's functioning (APA, 2013), ASD is defined by qualitative impairments in social and communication skills, as well as a restricted, rigid and obsessive pattern of interest and behavior (WHO, 1992; APA, 2013). ASD comprises a group of heterogenous disorders with features ranging from mild to severe forms of symptoms (APA, 2013). A child with ASD has difficulty in interacting and communicating with others. Typically, individuals with autism retreat into isolated activities or get fixated on a word or object. Core symptoms of autism are often accompanied by abnormalities in cognitive functioning, learning, attention and sensory processing. Symptoms are typically present before the age of 3 years (APA, 2013); symptoms could manifest during infancy while, in other situations, development would be normal in the first 1 year or more (Yeargin-Allsop *et al*. 2003; Klin *et al*. 2004). Compared with other childhood medical conditions, ASD is grossly neglected and less researched among African children living in the continent.

Fraught with uncertainties, ASD remains a disorder of obscure etiology. At present, there appears to be no definite biological marker for ASD. Of the three core areas of impairments, social skill impairment, such as avoidance or poor eye contact, difficulty in mixing with peers and lack of emotional reciprocity, remains the hallmark feature of the most severe and commonest form of ASD termed autistic disorder (APA, 1994). Across the globe, there are reports of a rapidly growing number of children being diagnosed with ASD, which has led to increased attention on impairments and diagnosis of the spectrum of disorders especially in the Western countries (Yeargin-Allsop *et al*. 2003; Chakrabarti & Fombonne, 2005; Mandell *et al*. 2005; MMWR, 2012). Despite the increase in the reported prevalence of the disorders, ASD remains poorly recognized, under-diagnosed and less researched (Mandell *et al*. 2005; Wiggins *et al*. 2006).

A search of literature reveals that most studies on ASD emanate from the West, while the topic is disproportionately neglected in African countries in spite of evidence that most children of the world live in the highly disadvantaged low- and middle-income countries (LMICs) of Africa (UNDP, 2004). Early intervention has been consistently associated with improved outcome for which early age at diagnosis is pivotal. Although the average age at diagnosis for ASD range from 3 to 6 years (Chakrabarti & Fombonne, 2001; Mandell *et al*. 2005), there were reports of a reduction in the mean age at diagnosis and that diagnosis of ASD can be made accurately in the first 2 years of life (Howlin & Moore, 1997; Charman & Baird, 2002; Yeargin-Allsop *et al*. 2003; Klin *et al*. 2004). This observation could be as a result of improved public and physician level of awareness with the inexhaustive and ever-expanding body of knowledge about ASD in the Western World.

Studies conducted among Africans living outside the African continent indicate that African children are more likely than Caucasian children to have a late diagnosis of ASD (Mandell *et al*. 2002, 2007, 2009). Often, African children receive wrong psychiatric diagnoses and spend longer duration in search of help before receiving definitive diagnoses (Mandell *et al*. 2007; Bello-Mojeed *et al*. 2010, 2012; Bakare & Munir, 2011*a*, *b*). While most available data on this topic emanated from the West, there is a dearth of information on the pattern of impairments and diagnosis of ASD among affected children living in African the continent. Early diagnosis with active early intervention has been identified to improve outcome in affected children. This study assessed the pattern of autistic impairments and age at diagnosis of ASD in a clinical population of Nigerian children with the spectrum of disorders.

## Methods

### Location

This study was conducted at the neurodevelopmental disorder clinic of the Child and Adolescent Mental Health Service Unit of Federal Neuro-Psychiatric Hospital (FNPH), Lagos, Nigeria. The child clinic is located within the community and serves as a walk-in as well as formal referral facility to children with varying mental health problems. Formal referrals are usually received from schools, general physicians/private hospitals and other specialist hospitals while informal referrals originate from family/relatives, friends and neighbors.

For this clinic, there is no waiting list for a client to get the first appointment. All cases presenting at the clinic for the first time are seen same day and scheduled for follow-up clinic appointment within the duration of few days to 2 weeks based on the clinical assessment of the individual client. This Clinic is the largest Child facility, in term of structure and personnel that renders services to children with neurodevelopmental disorders in Nigeria; serves majorly children in Lagos and gets the referral from all parts of the country with diversity in ethnic representation and socio-economic status.

### Ethical approval

Ethical approval was obtained from the ethical and research committee of FNPH, Lagos. Written consent was obtained from all mothers of children recruited for the study after explanations on the aims and objectives of the study were given to them.

### Participant and sampling method

Study participants comprise 60 children with ASD attending the study location at the time of the study. DSM-V criteria were used to make a diagnosis of ASD, while a symptom checklist was used to determine the pattern of impairments in each child with ASD. Children with ASD that met inclusion criteria were consecutively recruited into the study after obtaining written consent from their mothers.

### Material and procedure

A socio-demographic questionnaire and a symptom checklist for Autism were used for data collection.

### Socio-demographic and clinical questionnaire

The socio-demographic and clinical questionnaire was used to obtain data on the socio-demographic characteristics and clinical data of the study participants. Items in the socio-demographic questionnaire include the child's age, gender, birth order and level of education. Information was also obtained on child's age at the parental observation of ASD symptoms, at contact with specialist facility and at diagnosis.

### Symptom checklist for autism

The Symptom Checklist for examining the pattern of impairments in ASD was developed using items in the DSM-IV (Autistic Disorder, Aspergers and Pervasive Developmental Disorders NOS), DSM-V and ICD-10 criteria. It consists of 20 symptoms of ASD. The checklist assesses for presence or absence of varying symptoms of autism in the three core areas of impairments (social, communication and behavioral). Present in the Checklist is seven symptoms on the social impairment domain, five symptoms on the communication domain and eight symptoms on the behavioral domain. Responses on the Checklist were rated as either Yes (1 point) or No (0 points). The checklist was administered to mothers of children with autism who responded to the questions on behalf of their children. The Autism Symptom checklist was used to assess for the pattern of autistic impairments in the affected children. Two weeks test–retest reliability for the checklist was 0.96 with a Cronbach alpha of 0.91 (*p* < 0.001).

The study instruments were translated into Yoruba by a Yoruba-speaking psychiatrist and a linguist. The back translation was performed independently by another Yoruba speaking Psychiatrist and a Linguist. The back translation was then compared with the original translation by an independent panel and confirmed to be satisfactory before use.

The diagnosis of ASD was made based on DSM-V criteria. Two stages were involved in making a clinical diagnosis. The first stage involved comprehensive clinical assessment on every child with a previous diagnosis of ASD and any new patient suspected to have the spectrum of disorder; carried out by the first author who is a child psychiatrist with training in child development. The second stage comprised making or re-confirming a clinical diagnosis of ASD; the diagnosis of autism was made in any child who met the DSM-V criteria for the disorder before being recruited as study participants.

The mother completed the socio-demographic and other instruments. The literate mothers self-completed the socio-demographic questionnaire on behalf of the child. For the illiterate mothers, the questionnaires were read out to them, while the interviewer ticked off the items for them.

### Data analysis

The data collected were analyzed using the Statistical Package for Social Sciences (SPSS 16) software. Frequencies, proportions, means and s.d. were calculated. Group comparison of categorical variables was by chi-square (*χ*^2^). The entire tests were two-tailed and the level of significance was set at *p* < 0.05.

## Results

Sixty-two mothers of children with ASD were approached for recruitment of their children in the study but 60 children with a diagnosis of ASD participated in the study giving a response rate of 96.8%. Two mothers declined participation in the study due to a challenging behavior exhibited by one of the children difficult to manage by the mother who needed respite, while the second mother had to leave the clinic early to attend to her younger children at home. There was no difference in the socio-demographic characteristics of the children excluded and the sample recruited for the study.

### Socio-demographic characteristics of children with ASD

Table 1 shows the socio-demographic characteristics of children with ASD. Their ages ranged from 2 to 17 years with a mean age of 9.45 ± 4.33 years. The majority of the children (75%) were 12 years or younger and there was a preponderance (70%) of males. Most children were from monogamous (95.0%) Christian (78.3%) family, reside in an urban setting (66.7%) and from a middle socio-economic group (51.7%).

**Table 1.** Socio-demographic characteristics of children with ASD (n = 60)

Variables	Frequency (%)
Age range	Mean (s.d.)
2–17 years	9.45 (4.33)
Sex	
Males	42 (70.0)
Females	18 (30.0)
Family type	
Monogamous	57 (95.0)
Polygamous	3 (5.0)
Residence	
Urban	40 (66.7)
Semi-urban	17 (28.3)
Rural	5 (5.0)
Socio-economic class	
High	9 (15.0)
Middle	31 (51.7)
Low	20 (33.3)
Educational level	
None/out of school	25 (41.7)
Nursery/play group	24 (40.0)
Primary	10 (16.6)
Secondary	1 (1.7)
Birth order	
1st child	23 (38.3)
Middle child	19 (31.7)
Last child	18 (30.0)

### Health-contact and identification characteristics in ASD

Table 2 shows that about a half of the cases (51.7%) were referred from schools while the least proportions (16.7%) originated from informal sources including family, friends and neighbors. By the time each child attained the age of 2 years, all mothers (100%) of the children had observed an abnormality suggestive of ASD in their children but only 38.3% of them had contacted a child specialist clinic by the time the children were 6 years of age. Slightly above 33.3% of the children had a diagnosis of ASD made in the first 6 years of life (Table 2).

**Table 2.** Health-contact and identification characteristics of children with ASD (n = 60)

Variables	Frequency (%)
Referral source	
Parents/family	2 (3.3)
Friends/neighbors	8 (13.3)
General practitioners/private hospitals	5 (8.3)
Specialist hospitals	14 (23.3)
Schools	31 (51.7)
Age (months) abnormality observed	(Mean ± s.d. = 17.02 ± 6.78)
0–12 months	25 (41.7)
13–24 months	35 (58.3)
>24 months	0 (0.0)
Age (years) at contact with specialist clinic	(Mean ± s.d. = 8.13 ± 3.98)
6 years and below	23 (38.3)
7–12 years	27 (45.0)
13 years and above	10 (16.7)
Age (years) at diagnosis	(Mean ± s.d. = 9.0 ± 4.30)
6 years and below	20 (33.3)
7–12 years	26 (43.3)
13 years and above	14 (23.3)
Parent heard of ASD before diagnosis	
Yes	2 (3.3)
No	58 (96.7)

### Pattern of impairments in ASD

All the children with ASD (100%) exhibited poor eye contact, difficulty in mixing with other children and inability to consistently respond to his/her name. More than a half of them (55%) lack verbal communication (Table 3). The proportions of impairments that were not common are in the areas of object attachment (20.0%), odd postures (26.7%) and inappropriate facial expression (30.0%).

**Table 3.** Pattern of autistic impairment in children with ASD

Impairments	%
Social interactions impairment	
1. Avoidance of/poor/no eye contact	100.0
2. Inappropriate facial expression	30.0
3. Sustained odd body postures	26.7
4. Inability to indicate needs by gesture	93.3
5. Difficulty in mixing with other children	100.0
6. Lack emotional reciprocity	95.0
7. Inappropriate object attachment	23.3
Communication impairment	
8. Lack of speech development	55.0
9. Inability to initiate/sustain conversation	43.3
10. Stereotype and repetitive use of speech/action	48.3
11. No consistent response to name	100.0
12. Lack varied play	91.7
Behavioral impairment	
13. Motor mannerism	66.7
14. Resists changes in routines	83.3
15. Extreme distress for no apparent reason	76.7
16. Crying tantrum	78.3
17. Apparent insensitivity to pain	60.0
18. No fear of real danger	46.7
19. Acting aggressively	83.3
20. Preoccupation with an interest of abnormal intensity	20.0

## Age at diagnosis in ASD

Table 2 shows a significantly low mean age at the parental concern in ASD (17.0 ± 6.7 months). The mean age at diagnosis of ASD was 9.0 ± 4.3 years. The mean time lag from parental concern of features of ASD to seeking specialist care is 85 months and to diagnosis is 91 months.

**Fig. 1.**
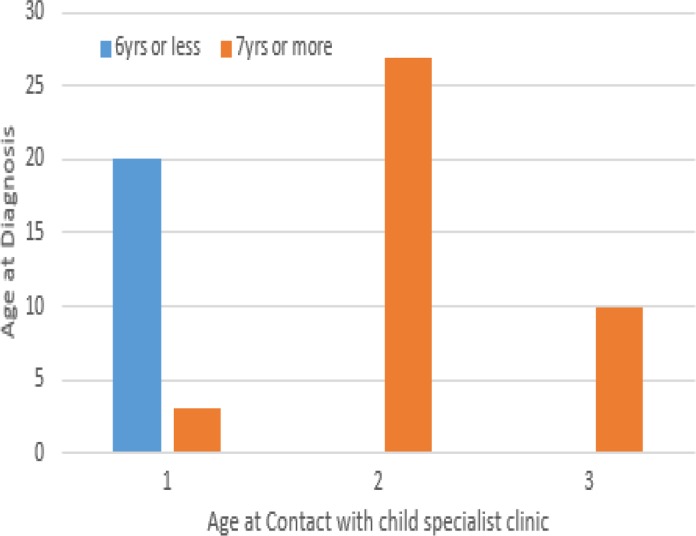
Bar chart showing age at contact with child specialist clinic (horizontal axis) against the age of diagnosis (vertical axis) at preschool and older/adolescent age group. On the vertical axis, (1) represents age range 6 years and below, (2) represents age range 7–12 years and (3) represents age range 13 years and above.

**Fig. 2.**
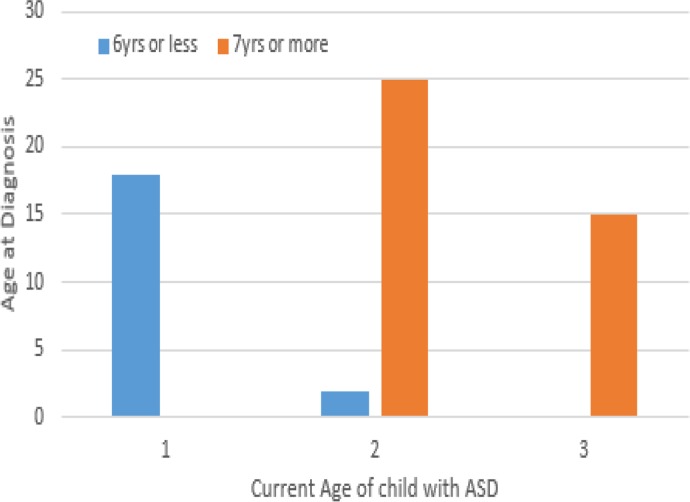
Bar chart showing current age of child with ASD (horizontal axis) against the age of diagnosis (vertical axis) at preschool and older/adolescent age group. On the vertical axis, (1) represents current age range 6 years and below, (2) represents current age range 7–12 years and (3) represents current age range 13 years and above.

Figures 1 and 2 illustrate the distribution of the age at diagnosis with current age of child and age at child's contact with the specialist clinic respectively. Relationships between age at diagnosis and socio-demographic characteristics are shown in Table 4. Compared to children with speech development, about three-quarter of those that were non-verbal (75.1%) received their first diagnosis at older age/adolescence, but the difference was not statistically significant (*χ*^2^ = 2.727, *p* = 0.110). Similarly, no statistically significant relationship was found between age at diagnosis and other symptoms of ASD in the studied sample.

**Table 4.** Associations between age at diagnosis and socio-demographic characteristics

	Age at diagnosis <7 years (preschool)	Age at diagnosis at ≥7 years (older/adolescents)	Total	
Variables	Frequency (%)	Frequency (%)	Frequency (%)	Difference
Age at 1st contact				
6 years and below	20 (87.0)	3 (13.0)	23	*χ*^2^ = 58.570, df = 2
7–12 years	0 (0.0)	27 (100.0)	27	*p* < 0.001
13 years and above	0 (0.0)	10 (100.0)	10	
Current age range				
6 years and below	18 (100)	0 (0.0)	18	*χ*^2^ = 51.667, df = 2
7–12 years	2 (7.4)	25 (92.6)	27	*p* < 0.001
13 years and above	0 (0.0)	15 (100.0)	15	
Gender				
Males	16 (38.1)	26 (61.9)	42	*χ*^2^ = 1.429, df = 1
Females	4 (22.2)	14 (77.8)	18	*p* = 0.232^a^
Educational level				
None/out of school	8 (32.0)	17 (88.0)	25	*χ*^2^ = 1.906, df = 2
Nursery/play group	10 (41.7)	14 (58.3)	24	*p* = 0.386
Primary/junior college	2 (18.2)	9 (81.8)	11	
Socio-economic class				
High	4 (44.4)	5 (55.6)	9	*χ*^2^ = 0.598, df = 2
Middle	10 (32.3)	21 (67.7)	31	*p* = 0.226
Low	6 (30.0)	14 (70.0)	20	
Residence				
Urban	17 (42.5)	23 (57.5)	40	*χ*^2^ = 4.538, df = 1
Rural & semi-urban	3 (15.0)	17 (85.0)	20	*p* = 0.044^c^
Autism awareness				
No	18 (31.0)	40 (69.0)	58	*χ*^2^ = 4.069,df = 1
Yes	2 (100.0)	0 (0.0)	2	*p* = 0.107^a^

a Fisher's exact value.

## Discussion

This study examined the pattern of impairments and age at diagnosis of ASD among a group of clinical population of children in Nigeria. The age range of 12 years and below for the majority of children with ASD (75%), seen in our study is similar to the findings of other studies carried out in child psychiatric clinics in Nigeria (Omigbodun, 2004; Aina *et al*. 2008). The finding of children in their early years of life is not unexpected as ASD is a medical condition of early onset. Also of note is the preponderance of males (70%) which is in keeping with findings of previous studies on a higher male to female ratio in autism occurrence (Chakrabarti & Fombonne, 2001; Yeargin-Allsop *et al*. 2003).

Although, most sampled children with ASD were referred through the school system, signifying the importance of the school system in the identification of children with ASD. However, the schools appear to lack basic skills required for early ASD identification as they were referred late and mostly had not been diagnosed with ASD prior to contact with the specialist center. An important finding in this study is a significantly low level of educational achievement among children with ASD. Only 1.7% proportion of those children with ASD was in the first year of a junior secondary school, on completion of a 6-year formal primary school education. In addition to the explanation of language impairment in especially the severe form of ASD, this finding is most likely a reflection of the challenges encountered in accessing appropriate education for this special group of disadvantaged African children (Omigbodun, 2004; Bello-Mojeed *et al*. 2010; Bakare & Munir, 2011*a*). In a study carried out in a child psychiatric clinic in Ibadan, Nigeria, Omigbodun (2004) found that 27.6% of the children in the study were out of school due to lack of suitable schools to meet their educational needs and children with autism were significantly associated with that problem. An interaction between severity of symptoms, associated intellectual impairment and ingrained stigma in Africa could also influence the observation of a low level of educational attainment among children with autism in our study (Aina *et al*. 2008; Bello-Mojeed *et al*. 2010, 2013, 2016; Bakare & Munir, 2011*b*). The previous report made in the country where the study was conducted, suggests that these children are often kept away from the stigmatizing community. Schools and the teachers present within may be reluctant to accept them due to a lack of knowledge about this disorder (Omigbodun, 2004; Bakare *et al*. 2009; Bello-Mojeed *et al*. 2010, 2013). Additionally, the lack of skills to tackle the disabilities associated with this disorder may also prevent mainstream schools from attending to the educational needs of children with ASD (Omigbodun, 2004; Bello-Mojeed *et al*. 2010, 2013).

The pattern of impairment reported in our study builds on previous evidence on impairments in three major areas of functioning in ASD (Kanner, 1943; Wing, 1969; Bartak & Rutter, 1974; Marchant *et al*. 1974; Chakrabarti & Fombonne, 2001; Yeargin-Allsop *et al*. 2003). The observation of more impairment in the social domain in this study is similar to the findings of studies conducted outside the African continent (Lord, 1995; Cox *et al*. 1999; Stone *et al*. 1999). Though social impairment is more specific to ASD and appears before the age of 3 years they could escape recognition in the context of the poor level of knowledge about features of ASD as reported earlier in Nigeria (Bakare *et al*. 2009; Bello-Mojeed *et al*. 2010, 2013).

Similar to our finding, previous studies reported a lack of ‘eye-to-eye gaze’ to be an important characteristic of ASD (Sorosky *et al*. 1968; Wing, 1969). Affected children often show a marked delay in both verbal and non-verbal communication skills as reported in the current study. The finding of a 55% rate of non-verbal communication among the children with ASD in the study location supports earlier reports of overrepresentation of non-verbal cases of ASD in Africa (Lotter, 1980; Belhadj *et al*. 2006; Mankoski *et al*. 2006; Bello-Mojeed *et al*. 2010, 2012; Bakare & Munir, 2011*a*). It has been suggested that about one-third to a half of individuals with autism failed to develop sufficient speech required for their daily communication needs (Noens *et al*. 2006). While many of them failed to acquire meaningful speech, ‘echolalia’, in which the child displays mechanical repetition of words or phrases spoken out by other people, often accompanied by pronominal reversal such as the use of ‘You’ instead of ‘I’ could be characteristic of their communication pattern. Many of the features found in our study, such as ‘difficulty in mixing with peers, gaze avoidance, difficulty in changing routine and lack varied play’, fit into Kanner's description of ‘infantile autism’. Assessment and subsequent diagnosis may be delayed in such children till attainment of school-age period (Yeargin-Allsop *et al*. 2003) when difficulties with peer interactions become apparent.

Although all mothers of children with the disorder had observed an abnormality suggestive of ASD in their children before the age of 2 years, the diagnosis of ASD was not made until a mean age of 9 years. A significantly high mean age of 9 years for ASD diagnosis found in this study demonstrates late diagnosis of the disorder among Nigerian children. Our report of maternal concern about features suggestive of ASD in their children before the attainment of age 2 years is similar to reports of studies emanating from the Western culture (Frith & Soares, 1993; Wiggins *et al*. 2006). On the other hand, the observation from the current study that children with ASD were not evaluated by a qualified professional until a mean age of 8.13 years, despite the presence of parental concern before the age of 2 years, is in contrast to research findings from outside Africa. Wiggins *et al*. (2006) in a study conducted among children with ASD in Atlanta reported a mean age of 4 years for evaluation by a qualified professional after parental concern in the age range from 12 to 21 months. Among possible factors, tortuous pathway to care in ASD, effect of perceived stigma on the affected child and parents, scarcity of ASD-specific intervention facilities in Africa could account for the marked delay in accessing professionals help (Omigbodun, 2004; Bello-Mojeed *et al*. 2010, 2012; Bakare & Munir, 2011*a*, *b*).

The finding of a mean age of 9 years for ASD diagnosis in our study is significantly higher than research findings of mean ages of 3.0–6.0 years among children with ASD from the West (Chakrabarti & Fombonne, 2001; Mandell *et al*. 2002, 2005; Yeargin-Allsopp *et al*. 2003). In a study conducted in the USA, Yeargin-Allsopp *et al*. (2003) found the mean age at first diagnosis of ASD in the studied children to be 3.9 years, while in a subsequent study in Pennsylvania Yeargin-Allsopp *et al*. (2003) observed that the average age at diagnosis for autistic disorder was 3.1 years. These findings suggest that over time, there appears to be a reduction in the age at which children with autism get diagnosed in the developed countries (Howlin & Moore, 1997; Yeargin-Allsop *et al*. 2003). On the contrary, the current study found a significant delay of about 7 years between the average time of parental observation of autistic features and diagnosis. The time lag between parental observation and diagnosis of ASD represents a vital period for institution of early intervention, which was lost by the child, family and the therapist. In the Western world, several programs are put in place to enhance early diagnosis and intervention in ASD. Comparatively, children with ASD living in an African country such as Nigeria are at a disadvantage not only in term of late identification, but also have to battle with the scarcity of ASD specific intervention facilities in the continent (Bello-Mojeed & Bakare, 2013; Bello-Mojeed *et al*. 2016). Research findings such as from the current study are important in informing policy makers of the urgent need to attend to the specific educational, social and medical needs of Nigerian children with ASD.

Late age of presentation at ASD child specialist center and place of residence were observed to be significantly associated with age at which the sampled population of Nigerian children receives a diagnosis of ASD. Children who lived in the rural and sub-urban areas were found to be more likely to receive a diagnosis of ASD later than their counterparts in the urban areas. This finding is in support of previous studies indicating a scarcity of specific intervention for children with ASD in rural areas (Palmer *et al*. 2005). In contrast to the Western countries, children with ASD in sub-Saharan African settings are seriously underserved, especially in the resource-disadvantaged rural communities. The very few available child health care facilities are concentrated in the urban areas with associated bottleneck of poor access.

This study has a limitation of being hospital-based, which precludes generalization and parents’ responses on behalf of the children relied on the memory recall of the participating mothers, which may not be foolproof. Also, the study did not make use of standardized ASD instrument, which may make it non-feasible to compare findings of the study to similar previous studies. In the diagnosis of ASD in Nigeria and other sub-Saharan regions, there are major difficulties in importing standardized instrument from Western countries to African children as some of the contents in such tools are culturally biased. Additionally, there are immense disparities in access to the available Western autism diagnostic tools. For example validated diagnostic tools such as ADI-R (Autism Diagnostic Interview-Revised) and ADOS (Autism Diagnostic Observation Scale), are very costly, require extensive training, lengthy to administer and culturally biased, all of which create huge obstacles to the access and use of such tools, especially in LMICs of Africa where most studies in ASD are self-sponsored. Despite the limitation, a considerable sample size was obtained and ASD Diagnosis made with Diagnostic Manual of Mental Disorders, Fifth Edition. Our study ensures a comprehensive psychiatric assessment and confirmation of the presence of core autistic symptoms and pattern of impairments were carried out.

To the best of the authors’ knowledge, this is the first study to address age at diagnosis and pattern of impairment in children with ASD in Nigeria. The implications of our study are significant. This study points to the importance of early recognition of ASD, appropriate specialist evaluation for definitive diagnosis and an increased need for improved knowledge among parents and the public in Nigeria. Educational intervention programs to improve the level of awareness and aid early recognition of ASD should be implemented. Social skills education and communication skills, especially in a naturalistic environment, should be an integral component of intervention programs. There is a need for community studies in this less researched area of childhood neurodevelopmental disorders. More studies are needed to assess for severity of autistic impairments and its relationship to the burden of care in African family caregivers. Findings of such studies should help in sensitizing policy makers to the importance of giving better attention to ASD and substantiating the need to give quality and evidence-based support to individuals and families with a diagnosis of ASD in Nigeria.

## Conclusions

Core symptoms/impairments of ASD are present in Nigerian children but a late presentation and evaluation at specialist facilities with a significant delay from the time of parental symptom observation to a diagnosis of ASD are common findings. Early screening for ASD, prompt access to needed specialist evaluation for definitive diagnosis, improved education/knowledge about ASD among parents and the public should be integral components of management of ASD in Nigerian children. Factors associated with late age at diagnosis of ASD should form an essential component of interventions and support services in the management of children with ASD in sub-Saharan Africa.
